# Optimizing the patient journey: Insights from early implementation of long‐acting cabotegravir and rilpivirine in four urban Ryan White‐funded clinics in the United States

**DOI:** 10.1002/jia2.70082

**Published:** 2026-02-12

**Authors:** Xavier A. Erguera, Mollie B. Smith, Priyasha Pareek, Alicia Dawdani, Kaylin V. Dance, Ryan S. Walker, Janet Grochowski, Jon Oskarsson, Matthew D. Hickey, Mallory O. Johnson, John Sauceda, Jose I. Gutierrez, Elizabeth T. Montgomery, Jonathan A. Colasanti, Lauren F. Collins, Moira C. McNulty, Kimberly A. Koester, Katerina A. Christopoulos

**Affiliations:** ^1^ Department of Medicine University of California San Francisco San Francisco California USA; ^2^ University of Massachusetts Chan Medical School Worcester Massachusetts USA; ^3^ Department of Medicine Emory University Atlanta Georgia USA; ^4^ Department of Medicine University of Chicago Chicago Illinois USA; ^5^ Ponce de Leon Center Grady Health System Atlanta Georgia USA; ^6^ Columbia University Vagelos College of Physicians and Surgeons New York New York USA; ^7^ Family Health Care Nursing University of California San Francisco San Francisco California USA; ^8^ Research Triangle International Berkeley California USA; ^9^ Department of Epidemiology and Biostatistics University of California San Francisco San Francisco California USA

**Keywords:** anxiety, cabotegravir, HIV acquisition, pain management, patient‐centred care, rilpivirine

## Abstract

**Introduction:**

Long‐acting injectable (LAI) cabotegravir/rilpivirine (CAB/RPV) represents a breakthrough in HIV treatment. However, understanding how to optimize real‐world service delivery and user experiences among people with HIV (PWH) remains limited.

**Methods:**

Between August 2022 and December 2024, we conducted semi‐structured interviews with PWH at four academic Ryan White‐funded HIV clinics in Atlanta, Chicago and San Francisco. Eligibility criteria were current LAI‐CAB/RPV use with ≥3 injections or having discontinued. Interviews were analysed using thematic analysis grounded in descriptive phenomenology.

**Results:**

Among 69 participants, 48 of whom were receiving LAI‐CAB/RPV and 21 who had discontinued, we identified three themes that highlighted opportunities to enhance patient‐centred service delivery of LAI‐CAB/RPV: (1) enhancing knowledge and self‐efficacy in using oral antiretroviral therapy (ART) in cases of missed or late injections; (2) improving patient comfort and confidence, particularly regarding injection anxiety, pain management and blood draws; (3) attending to the potential evolution of patient‐provider relationships, as in most cases injection visits outnumber primary care visits. In addition, PWH may experience depressive feelings upon discontinuation, even if they view it as the right decision.

**Conclusions:**

Optimizing the LAI‐CAB/RPV patient journey requires developing a specialized support framework that is distinct from oral ART protocols. This new treatment modality requires a tailored approach that addresses unique challenges, including facilitating candid discussions about adherence contingencies, managing the physical and psychological aspects of injection experiences, ensuring meaningful and consistent provider relationships amid changing care patterns and providing enhanced support during treatment transitions.

## 1 INTRODUCTION

Long‐acting injectable (LAI) cabotegravir/rilpivirine (CAB/RPV), given as two intramuscular injections every 4 or 8 weeks, depending on the patient's treatment plan, is a paradigm shift in HIV treatment as the first and currently only complete injectable antiretroviral therapy (ART) regimen [1, 2, 3]. If more than 7 days late for the scheduled injection, it is recommended to resume oral ART until the next injection is received to maintain viral suppression (also known as oral bridging therapy). If oral therapy is not feasible, CAB/RPV injections may still be resumed under clinical guidance after reassessing viral suppression status and the duration since the last dose [4].

In studies evaluating early adopters of LAI‐CAB/RPV, many people with HIV (PWH) reported significant benefits, including liberation from daily pill regimens [5, 6], increased privacy and convenience [7, 8], and, for those with adherence challenges, improved self‐worth and care engagement upon achieving viral suppression [9]. However, barriers to successful LAI‐CAB/RPV use include injection‐related pain and anxiety [10], medical mistrust [11] and the need for more frequent clinic visits [12, 13].

While the psychosocial benefits of LAI‐CAB/RPV among PWH have been elucidated [9], less is known about how best to optimize service delivery of LAI‐CAB/RPV in real‐world settings from the patient perspective. We sought to understand early adopters’ experiences with LAI‐CAB/RPV, both from those who had continuation and discontinuation experiences, in order to identify opportunities for improving patient‐centred delivery of this novel treatment modality.

## 2 METHODS

This analysis leveraged data from a cross‐sectional qualitative study consisting of semi‐structured interviews with 59 early adopters of LAI‐CAB/RPV collected between August 2022 and May 2023 at four academic Ryan White‐funded HIV clinics in San Francisco (Ward 86), Chicago (the University of Chicago) and Atlanta (Emory Midtown; the Ponce de Leon Center at Grady) [9]. Early adopters refer to patients receiving HIV primary care and being the first non‐research individuals to access this treatment following U.S. food and drug administration (FDA) approval. Between June and December 2024, we conducted an additional 10 semi‐structured interviews at Ward 86 with individuals who had discontinued LAI‐CAB/RPV. These interviews were added to strengthen the understanding of discontinuation experiences, an area where saturation was not reached in the original dataset due to the small number of early discontinuers during the initial data collection period. Although conducted approximately 1 year after the early adopter phase, these interviews provide complementary insights into the reasons for and contexts surrounding discontinuation as the programme matured. While each clinical site had slightly different delivery models for LAI‐CAB/RPV, all clinics incorporated pre‐injection counselling provided by clinic pharmacists or nursing staff in addition to HIV provider consultation [14, 15, 16]. The administration of injections was primarily performed by trained nursing staff, though FDA labelling permits other qualified healthcare professionals (i.e. pharmacists, physicians) to deliver the injections. Key differences between sites included workflow organization, staffing models and scheduling approaches [14, 15, 16, 17].

The core research team included a physician lead at each site (KAC, MCM, JAC and LFC); a medical anthropologist (KAK); research coordinators with qualitative experience (XAE, MBS, RSW, KVD and AD); and a third‐year medical student (PP). Guided by a socio‐ecological model of care engagement [18], the interview guide explored participants’ experiences living with HIV and taking oral ART; motivations for initiating CAB/RPV‐LA; the process of starting and receiving injections; and reflections on CAB/RPV‐LA use compared with oral ART. When applicable, interviews also addressed reasons for discontinuation and transitions to alternative HIV treatment regimens, including treatment interruption. The guide was piloted with three patients to assess content, flow and richness of responses, and was revised iteratively.

Eligibility criteria included: (1) age ≥18 years; (2) having received ≥ 3 injections or received at least one injection and discontinued; and (3) fluency in English. Interviews were conducted either in‐person at the clinic or via video conference, based on the participant's preference, and were audio‐recorded. Interviews lasted 60–90 minutes and were professionally transcribed. After each interview, participants completed a brief survey capturing demographic and psychosocial characteristics (e.g. history of psychiatric illness, housing instability, use of methamphetamines and injection drug use). Chart abstraction supplemented these data with key clinical information (e.g. HIV‐1 viral load at initiation). A detailed description of the qualitative methods for the original dataset (*n* = 59), including recruitment, data collection and familiarization/codebook development, has been published elsewhere [9].

For this analysis, we conducted a three‐stage thematic approach grounded in descriptive phenomenology [19]. As noted, the *familiarization* phase occurred in the context of prior analysis [9], while the *exploration* and *recontextualization* phases are unique to this analysis.

In the *exploration* phase, we utilized the codebook developed during the parent analysis, leveraging Dedoose (version 9.054) software. Two coordinators coded each transcript, resolving differences through discussion. XAE reviewed all excerpts from the early adopter interviews (*n* = 59) previously coded as “uptake and considerations” and “LAI‐CAB/RPV experience,” focusing on areas for improvement in LAI‐CAB/RPV implementation. A more refined set of codes was subsequently developed based on this review, including adherence support, emotional support, pain management, patient education and workflow issues. XAE re‐coded the original dataset using these refined codes, summarized the resulting excerpts and generated a memo for each code. These memos were used to identify preliminary themes, which were shared with and revised by the core analysis team (KAC and KAK).

Two analysts (XAE and KAC) read and discussed the 10 additional interviews with participants who had discontinued LAI‐CAB/RPV. XAE organized the data into a structured table, capturing aspects of key research interest: initial appeal of LAI‐CAB/RPV, reasons for discontinuation, decision‐making process, role of primary care provider and attitudes post‐discontinuation. Two analysts (XAE and KAC) reviewed the analytic table to determine whether cases were adding new information or aligning with previously identified topics of importance, thereby aiding in the determination of saturation. XAE subsequently wrote a memo summarizing the alignment of these data with the aforementioned preliminary themes and highlighting new insights.

The *recontextualization* phase involved re‐reading full transcripts from the combined datasets (*n* = 69) to situate themes within individual narratives, examining variations by site, viraemic status and discontinuation experience, resulting in an analytic memo that refined the insights reported herein. Participant quotes are attributed by age, race, gender, clinic site, viral suppression status at LAI‐CAB/RPV initiation (VS = virally suppressed; NVS = not virally suppressed) and whether the participant continued (CONT) or discontinued (DISC) treatment.

The University of California, San Francisco Institutional Review Board served as the single IRB and approved this research. Written informed consent was obtained from all participants prior to interviews.

## 3 RESULTS

### 3.1 Study population

The 69 participants included 48 PWH currently using LAI‐CAB/RPV and 21 who had discontinued (Table 1). Ages ranged from 25 to 73 years (median: 49), with 46 (67%) men, 30 (43%) Black or African American and 10 (14%) Latine. Thirty‐three (48%) disclosed a history of psychiatric conditions (e.g. depression, anxiety, bipolar disorder and schizophrenia). Housing instability affected 19 (32%) individuals, while 28 (41%) reported financial hardship. Additionally, 16 (23%) participants acknowledged methamphetamine or injection drug use within 30 days preceding study participation. Participants had a median of 7 injection visits (IQR: 4–10) over a median of 224 days (IQR: 140–329). Each visit involved two intramuscular injections—one cabotegravir and one rilpivirine—administered at the same appointment.

**TABLE 1:** Characteristics of participants receiving long‐acting injectable cabotegravir/rilpivirine in routine care

	Persisted	Discontinued	Total
	*n* = 48 (%)	*n* = 21 (%)	*n* = 69 (%)
Site^a^			
Ward 86	20 (42%)	15 (71%)	35 (51%)
Ponce De Leon	8 (17%)	2 (10%)	10 (15%)
Midtown	7 (15%)	3 (14%)	10 (15%)
University of Chicago	13 (27%)	1 (5%)	14 (20%)
Age, median (min/max)	50 (26−73)	48 (25−68)	49 (25–73)
Age^a^			
18–29 years	5 (10%)	3 (14%)	8 (12%)
30–49 years	18 (38%)	10 (48%)	28 (41%)
≥50 years	25 (52%)	8 (38%)	33 (48%)
Time period of diagnosis^a^			
After 2021	3 (6%)	2 (10%)	5 (7%)
2007–2020	21 (44%)	10 (48%)	31 (45%)
1997–2006	10 (21%)	5 (24%)	15 (22%)
Before 1997	14 (29%)	4 (19%)	18 (26%)
Gender^a^, ^b^			
Cisgender male	29 (60%)	17 (85%)	46 (68%)
Cisgender female	14 (29%)	1 (5%)	15 (22%)
Transgender woman	4 (8%)	1 (5%)	5 (7%)
Other^c^	1 (2%)	1 (5%)	2 (3%)
Race^b^			
White	15 (31%)	7 (35%)	22 (32%)
Black	26 (54%)	4 (20%)	30 (44%)
Multiracial/other	7 (15%)	9 (45%)	16 (24%)
Hispanic ethnicity^b^	4 (9%)	6 (29%)	10 (15%)
Heterosexual^b^	21 (45%)	4 (19%)	25 (37%)
Housing^a^			
Own/rent	34 (71%)	16 (76%)	50 (72%)
Single room occupancy/hotel	9 (19%)	2 (10%)	12 (17%)
Transitional/treatment programme	0 (0%)	1 (5%)	1 (1%)
Staying with friends/family	4 (8%)	2 (10%)	6 (9%)
Homeless	1 (2%)	0 (0%)	0 (0%)
Reported methamphetamine or IDU in past 30 days^b^	8 (17%)	8 (40%)	16 (24%)
Self‐reported mental health diagnosis	24 (50%)	9 (43%)	33 (48%)
Education^a^, ^b^			
At least some college/post‐graduate studies	32 (68%)	15 (71%)	47 (69%)
High school/General Education Development Test	10 (21%)	3 (14%)	13 (19%)
Less than high school	5 (11%)	3 (14%)	8 (12%)
Financial situation^a^, ^b^			
Comfortable, have money for extras	9 (20%)	2 (10%)	11 (16%)
Have money for necessities	16 (35%)	12 (57%)	28 (42%)
Barely paying the bills	12 (26%)	3 (14%)	15 (22%)
Struggling to survive	9 (20%)	4 (19%)	13 (19%)
History of incarceration^b^	18 (38%)	8 (38%)	26 (38%)
Reported use of other daily oral medications	32 (67%)	15 (71%)	47 (68%)
CAB/RPV‐LA initiation with plasma HIV‐RNA >50 copies/ml	8 (17%)	10 (48%)	18 (26%)
Log^10^ plasma HIV‐RNA in those viraemic at CAB/RPV‐LA initiation, mean (SD)	4.00 (1.60)	4.55 (0.83)	4.25 (1.31)
CD4 cells/mm^3^ at CAB/RPV‐LA initiation, median (IQR)			
Viraemic at initiation (HIV‐RNA >50 copies/ml)	475 (85, 651)	262 (54, 746)	361 (56, 746)
Virally suppressed at initiation (HIV‐RNA <50 copies/ml)	693 (457, 896)	575 (555, 753)	680 (460, 889)
CAB/RPV‐LA dosing interval^a^, ^b^			
Every 4 weeks	18 (38%)	18 (86%)	36 (52%)
Every 8 weeks	13 (27%)	1 (5%)	14 (20%)
Started every 4 weeks and transitioned to every 8 weeks	17 (35%)	2 (10%)	19 (28%)
Number of injection visits at interview, median (IQR)^d^	7 (4, 10)	4 (3, 6)	6 (4, 9)
Self‐reported late injections^b^	4 (8%)	2 (10%)	6 (9%)
Plasma HIV‐RNA <50 copies/ml at interview^b^	48 (100%)	19 (95%)	67 (99%)
Reasons for discontinuations			
Virologic failure	N/A	4 (19%)	4 (6%)
Patient preference	N/A	17 (81%)	17 (25%)

*Note*: All participants initiating CAB/RPV‐LA with plasma HIV‐RNA levels>50 copies/ml were on a q4‐week dosing regimen, except for two. All participants who discontinued CAB/RPV‐LA were on a q4‐week dosing regimen, except for three.

Abbreviations: CAB/RPV‐LA, long‐acting cabotegravir/rilpivirine; HIV‐RNA, human immunodeficiency virus ribonucleic acid; IQR, interquartile range; SD, standard deviation.

^a^ Totals may not equal 100% due to rounding to the nearest whole number.

^b^ Data missing for gender (*n* = 1), race (*n* = 1), ethnicity (*n* = 2), sexual orientation (*n* = 1), drug use (*n* = 2), education (*n* = 1), financial situation (*n* = 2), incarceration history (*n* = 1), self‐reported late injections (*n* = 13) and plasma HIV RNA at interview (*n* = 1).

^c^ Other gender identities: gender‐queer and “male‐ish.”

^d^ Each visit involved two intramuscular injections—one cabotegravir and one rilpivirine—administered at the same appointment.

### 3.2 Findings

Three themes emerged regarding programmatic features that are important to consider with long‐acting ART: (1) providing education on and clear plans for using oral ART as a bridge with late injections; (2) improving patient comfort and confidence, particularly regarding pain management and anxiety around injections and blood draws, possibly with peer support; and (3) attending to the potential evolution of patient‐provider relationships, as injection visits outnumber primary care visits. In addition, some who discontinued injections voiced feelings of depression, highlighting the role for enhanced psychological support and monitoring from the care team during this time.

### 3.3 Providing education on and clear plans for using oral ART with late injections

We asked participants about their understanding of what to do in the event of a missed or late injection. Patient understanding of missed injection protocols was often vague. Many relied on incomplete information or assumptions. “*What happens if I miss an injection? I don't know because I haven't missed an injection yet. I guess I would talk to my doctor and find out what to do*.” *(30YO Latine Trans Woman, SF, VS, CONT)* Some were aware of a brief “grace period”—often cited as 5−7 days—but they remained unsure of the steps to take if they missed this window entirely. “*They said seven days before or seven days after… I'm not sure what they told me about if I missed it…*.” *(28YO Black Man, ATL, VS, DISC)* In contrast, participants who had been late for or rescheduled appointments had a clearer understanding, often gained through experience rather than guidance. “*If I miss an appointment, I just try to reschedule it within that time…if it's over the period of time, I go back to taking my other meds*.” (*51YO White Man, SF, VS, CONT)*

Many participants expressed an unwavering commitment to attending their injection appointments on time. Ironically, their commitment might have inhibited seeking clarification about the role of oral ART in missed or late injections. “*I've never missed an appointment, never been late…I want to show that I'm taking it seriously…*” *(67YO White Man, SF, VS, CONT)* Some said they even avoided asking about missed injection appointments, fearing it might be seen as a lack of commitment. “*Interviewer: Do you know what the process would be if you were to miss an injection appointment? Informant: No, I don't…I don't want to find out either*.” *(40YO White Man, ATL, VS, CONT*)

Participants were asked to describe how their clinics communicated the risk of resistance associated with missed or delayed injections and how they understood and responded to that information. While participants generally recognized that late injections could lead to drug resistance, their understanding of this concept varied widely. Some expressed vague knowledge about how resistance develops. “*What I didn't want to happen is for it to reverse on me, and like, my body will completely reject it…I don't want to reach that point ever, because that takes me back to square one*.” *(43YO Man, Race Not Reported, SF, NVS, DISC)* For other participants, the threat of resistance took on a more catastrophic tone, where missing injections was viewed as a potential path to irreversible harm.

“If I start missing a dose, I will develop resistance. And when we have a problem down the road, it will be hard to find any kind of medication for me. And it's going to be fatal, like, none of the medications could work on me.” (51YO White Man, SF, NVS, CONT)

The availability of backup oral ART provided some reassurance to patients, though many reported insufficient guidance regarding its proper use, including the duration of use and protocols for transitioning back to LAI‐CAB/RPV.

“Informant: Yeah”, they gave me a thing of pills to take in case I miss one… Interviewer: When they gave you the backup pills, did they tell you when you would need to take them?

Informant*: Oh, “I have no idea.” (33YO Latine Woman, SF, VS, CONT)*

In contrast, some patients viewed these bridging pills as unnecessary, convinced that their commitment to attending appointments on time rendered the bridging pills irrelevant. “*Yeah, I still have some pills, but I don't take them because it's a must that I come to do what I got to do. So…I'm bringing my pills to give them back to the doctor*.” *(66YO Black man, CHI, VS, CONT)*

Participants who completed structured orientations, formal educational sessions provided by the clinic, demonstrated greater confidence in their knowledge of oral ART bridging. These orientations were typically organized by clinic staff and delivered in person or via video modules, ensuring that all patients received standardized information about the medication, its dosing and potential side effects. In some cases, participants were required to sign documentation confirming completion of the session. These structured orientations complemented other self‐directed learning activities, including independent research and consultations with healthcare team members such as primary care providers and pharmacists.

“It's just like a little class that you took. It was half a day, explaining the medication and the side effects and stuff like that…they make you sign a piece of paper saying that you watched the video…they told me everything.” (54YO Black Man, ATL, VS, DISC)

### 3.4 Enhancing patient comfort and confidence in long‐acting injectable treatment

Many participants described feeling nervous, scared or anxious leading up to and during their first injection, often due to fear of pain or uncertainty about the procedure.

“*Interviewer*:” How did your first injection appointment go?

Informant: I was kind of scared because they said it kind of hurt…Then they were having a hard time finding…exactly where the injection was supposed to go, so it was making me more anxious, like, ‘Oh my gosh, do these people know what they're doing?’ (30YO Latine Trans Woman, SF, VS, CONT)

For some, these feelings persisted with subsequent injections. Consistency of care providers, that is having the same nurse administer injections, helped build trust and reduce anxiety. “*The nurse who gives me my injection…is great. He doesn't know [it], but he takes a lot of stuff off my mind…[I] don't want to be here. But I…respect these people and let them…give [me] the best treatment*.” *(57YO Black Woman, CHI, VS, CONT)* Pre‐injection education and clear communication also reduced anxiety. Participants reported that they valued opportunities to ask questions and receive reassurance. “*Yep, the nurse that gave the injections. Like before he did the injections, I think I spent about 20 minutes asking questions…*” *(30YO Black Man, CHI, VS, CONT)* Regular follow‐ups from clinic staff also eased concerns. “*[The nurse] would always ask*, ‘*Hey, how was it*,’*…Or she'll call me a couple of days after….just to say, ‘Hey, are you experiencing any side effects*.’” *(43YO Latine Man, ATL, VS, DISC)*

Participants also noted several techniques that helped minimize injection site‐related pain, such as body positioning during injections. “*When I lay down and got the injections, I did not feel that sensation or that shock*.” *(43YO Latine Man, ATL, VS, DISC)* Hydration also seemed to help. “*I chugged a whole water bottle before I did it, and it helped a lot*.” *(28YO Latine Man, SF, NVS, CONT)* Distraction techniques also proved useful. “*She brought me like an ice pack to put on my chest. I guess it distracts the nerves or relaxes you or something. And then she told me to wiggle my toes*.” *(30YO Latine Trans Woman, SF, VS, CONT)* Post‐injection care strategies included warm baths, ice packs and staying active.

Interestingly, participants who initiated LAI‐CAB/RPV with viraemia (> 50 copies/ml) demonstrated greater tolerance for injection‐related challenges and discomfort. For these individuals, the decision to start injectable treatment was often framed as a critical health intervention by trusted providers. The seriousness of their health situation appeared to override concerns about injection site pain or administration processes.

“I was in a bad relationship with somebody and neglecting myself, my life, and my health. I stopped taking the HIV meds for quite a bit of time. So, my social worker was worried about [me]. And she told me about the shots…I hate needles. Like, I really, really don't like needles. But it's worth it… I can tolerate the little needle poke. But for 30 days, I have peace of mind.” (51YO White Man, SF, NVS, CONT)

As noted in a previous analysis by our team [9], pain related to injections and the anxiety of “gearing up” for injections were common reasons for discontinuation, as the burden of injections outweighed potential benefits, particularly for those without oral ART adherence challenges. Interviews with PWH who discontinued LAI‐CAB/RPV revealed more nuance in the conceptualization of injection burden, as several individuals noted that a cumulative fatigue from repeated exposure to needles, both through the two injections required for LAI‐CAB/RPV as well as blood draws at each injection early on, contributed to their sense of treatment burden related to too many “pokes.”

“…you've got to do the whole shebang. Like, blood draw, this. It's going to be a whole day…I refuse to be all drained and worn and torn…you're out of your mind….Regardless if it's about my health or not, that's a lot.” (40YO Multiracial Non‐Binary Person, SF, NVS, DISC)

Those who discontinued expressed interest in peer support programmes to help navigate treatment decisions and adverse effects, noting that peers could provide valuable real‐world perspectives and support not fully captured by clinical counsel alone.

*Interviewer: Do you have any suggestions for us for improving the program?*

Informant: I don't know if it's possible to be able to talk to someone who has gone through the process and is still actively taking Cabenuva, and also someone who's gone through it and decided to stop taking it…to hear from them firsthand what it's like, what the pain's like, what they like about it, what they didn't like about it….how they are able to cope with the pain….(51YO Asian Man, SF, VS, DISC)

### 3.5 Evolution of the patient‐provider relationship

Participants described primarily interacting with clinic‐based pharmacy and nursing staff for injection appointments, with nurses being the main point‐of‐contact. As a result, some participants saw their primary care provider less frequently. “*Interviewer: Do you typically see your doctor at your appointments? Informant: Every four to six months now…I probably had two or three treatments without seeing [my doctor]*.” *(36YO Black Man, ATL, VS, CONT)* Notably, among participants who discussed their primary care provider relationship, none reported deterioration in the relationship quality or dissatisfaction with reduced contact. In fact, most described their relationships with their primary care provider as unchanged despite fewer visits, and others reported even stronger bonds, rooted in increased trust related to treatment effectiveness. “*I feel closer with [my doctor]…I trusted him to give me the right meds and I feel better because of it*.” *(47YO White Genderqueer Person, SF, VS, CONT)*

While patients previously saw their primary care providers at all routine visits, they now have additional encounters with nursing staff for injection administration between their regular primary care provider appointments. This evolution yielded several important benefits. Informants voiced that injection visits strengthened relationships with nursing and pharmacy staff, and, in some cases, shifted traditional care dynamics by creating a broader support network and more frequent healthcare touchpoints. This shift has created a more distributed care network, with nurses taking on expanded roles beyond injection administration, including sexual health screenings, vaccinations and helping patients navigate medication concerns. Informants reported that primary care visits became more comprehensive by allowing primary care providers to focus on non‐HIV health matters. One patient described nurse‐led care as “more normal,” reflecting this shift in how routine HIV care is delivered and experienced.

“I'll see my doctor too if I need to. Because like I said, the last few times I've been there, I also had other things going on. But this last time was more normal. I didn't see my doctor at all. It was just the injection nurse and then the front desk staff.” (28YO Latine man, SF, NVS, CONT)

However, this evolution in care delivery was not without its challenges. While participants stated that they valued support from pharmacists and nurses, some voiced a desire for more contact with primary care providers early in the course of injectable treatment. As we previously described [9], one participant who discontinued LAI‐CAB/RPV after 5 months cited limited primary care provider contact, injection site reactions, weight gain and recurring depression as factors. Here, we further highlight that he expressed ambivalence about stopping injectables and the loss of supportive clinical relationships. His experience highlights the delicate balance in transitioning care responsibilities.

“Try not to avoid your patients more since they're on the [injectable]…Like, if they're on the [injectable], treat them just….as if they were on oral medications. Like, when I was on the oral medication, I was receiving lots of help, lots of counseling, everything. Once I went to Cabenuva, I basically lost touch with everyone.” (25YO Latine Man, CHI, VS, DISC)

Importantly, participants indicated that they appreciated when injection appointments were coordinated with primary care provider visits for convenience and continuity of care. These participants seemed to appreciate rather than complain about the longer time spent at the clinic to get both visits done.

“In fact, when I was here last week, I got the blood work done, I saw [my doctor], I got the Cabenuva, and I saw [the pharmacist] about a prescription. I got everything… I was very, very – very, very pleasantly surprised we were able to do it that way.” (67YO White Man, SF, VS, CONT)

Concerns about treatment efficacy emerged as a crucial area requiring ongoing primary care provider engagement. Patients expressed deep concerns about maintaining undetectable viral loads, seeking clear guidance about monitoring and potential complications. “*My biggest concern was that I go back to being detectable. I don't want to get sick*.” *(55YO Black Woman, SF, NVS, CONT)* Moreover, those who discontinued due to virologic failure or concerns about continued efficacy reported profound negative mental health impacts, particularly among those who had previously struggled with oral ART adherence.

“I just put myself in my room and stayed there for a good three or four days…I was so depressed about not taking it anymore…Cabenuva's not going to work for me. I can die now. A lot of negative thoughts went through my head.” (35YO Latine Man, SF, NVS, DISC)

“So, when they called and told me that I was resistant. I just kind of went into a depression because my whole thing was like I finally felt human again…And then they told me I was resistant, and it kind of shattered the glass. I was going to have to start on those damn pills again.” (49YO Black Man, SF, NVS, DISC)

These statements underscore the role for increased support and counselling from primary care providers following discontinuation. Even for those who believed discontinuation was the right decision for them to alleviate injection burden, scheduling challenges and side effects, support from a primary care provider was welcomed as a complement to support received from nurses, pharmacists and others.

## 4 DISCUSSION

This qualitative analysis is the first to focus on the patient experience of LAI‐CAB/RPV in a socio‐demographically diverse multisite group of PWH in routine care. Although it occurred in the United States, the findings reflect themes with broad relevance as health systems worldwide introduce or expand LAI‐ART. The shift from daily oral therapy to an injectable regimen represents not only a biomedical innovation but also a transformation in how PWH perceive their treatment, interact with their providers and exercise their sense of agency in care. Across diverse contexts, the principles of supportive, non‐judgemental communication (enabling open discussion about concerns, including late or missed injections), person‐centred care (attending to comfort, pain and emotional needs, potentially with peer involvement) and continuity in provider relationships (preserving trust even as care models shift) are foundational, particularly where structural barriers, workforce capacity and patient trust intersect.

Our findings revealed the importance of providing emotional and practical support—particularly through contact with one's care team and the anticipated or desired support of peers—before and after initiating injections to mitigate anxiety related to the injection process and concerns about treatment efficacy. This support was also welcomed in the context of discontinuation. In addition, we observed a surprising pattern between the history of oral ART/injection adherence and knowledge of late injection contingencies: PWH with excellent adherence to oral ART/injections had not internalized messaging around what to do in these situations and, in some cases, actively avoided asking questions about it. Further, our data highlighted a cautionary note regarding the potential for patients to feel more distanced from their primary care provider after initiating LAI‐CAB/RPV, despite frequent injection visits and stronger relationships with other members of the care team.

Injection‐related anxiety emerged as a prominent concern of PWH, particularly during treatment initiation, but also due to cumulative fatigue from injections and blood draws. Our findings revealed several promising strategies for addressing these concerns, including education on what to expect, consistency in who administers injections and guidance on specific pain management techniques. These findings align with lessons from the Whitman Walker pre‐exposure prophylaxis clinic regarding delivery of LAI‐CAB for HIV prevention, where spa‐like injection rooms not only reduced anxiety about intramuscular injections but also created comfortable, low‐stress environments that providers perceived improved adherence [20]. Moreover, we found that PWH initiating LAI‐CAB/RPV with viraemia due to adherence challenges expressed greater resilience with regard to injection‐related pain. While this resilience may increase provider comfort in prescribing to this group, it remains important to strike the right balance between emphasizing the potential benefit of LAI‐CAB/RPV for one's health and acknowledging its challenges, such that PWH do not minimize or discount treatment burdens [9].

Some participants expressed fears regarding treatment efficacy and the risk of virologic rebound. While formal clinical guidelines for managing viral load fluctuations in the context of LAI‐CAB/RPV are still evolving, these findings underscore the importance of proactive patient counselling. This should include educating patients about the possibility of viral load fluctuations, keeping them informed of their lab results and discussing potential next steps should unexpected results occur. Clinicians should acknowledge that some variation may be expected (e.g. viral blips even with on‐time injections), outline the circumstances that would warrant concern (e.g. sustained increases, detectable viraemia in the context of late injections) and establish clear communication channels.

Formalized education initiatives successfully boosted patient confidence in managing their treatment, including their understanding of oral ART bridging as a component of their care. The success of these programmes appears to stem from their comprehensive approach. Rather than relying on a single method of information delivery, these structured programmes incorporate various learning approaches (e.g. videos, brochures and consultation), making the information more accessible and memorable for different types of learners. The success demonstrated here suggests that investing in formal patient education is not just beneficial but perhaps essential for optimal treatment outcomes.

In addition, participants voiced the important role peer support could play in LAI‐ART initiation and decisions about persisting or discontinuing injection use. The value of peer support programmes lies in their unique ability to bridge the gap between clinical expertise and lived experience [21]. While healthcare providers offer essential medical guidance, peers bring an authenticity and relatability to treatment discussions that come only from walking a similar path [22]. Peers could share personal coping strategies, describe how their effects evolved over time and offer practical, day‐to‐day management tips drawn from their own experience. An effective approach could be to combine clinical counsel with peer support [23].

While healthcare providers need to ensure patients are ready and committed to LAI‐CAB/RPV, communicating that “perfect adherence” is the only acceptable standard may discourage important discussions. For example, questions about missed doses may indicate responsible treatment planning rather than a lack of commitment. Studies show 7–35% of PWH receiving LA‐CAB/RPV experienced delayed injections [14, 15, 16], highlighting the need for open dialogue about adherence challenges. While proactive communication about managing late or missed injections is important for clinical continuity, this approach should be tailored to the individual. For some participants who expressed strong commitment to their injection schedules, repeated emphasis on contingency planning could inadvertently signal mistrust in their reliability, potentially undermining motivation and partnership in care.

Our results also indicate that LAI‐CAB/RPV is inevitably changing relationships between patients and the HIV care team by shifting frequency in primary care provider visits to comparatively more interactions with nursing and pharmacy staff. While many PWH appreciate the convenience of quick injection visits, others reported feeling disconnected from their primary care providers, highlighting the importance of intentionally maintaining strong therapeutic relationships even as care models evolve. Encouraging integrated or “bundled” visits, whether with primary care providers or multidisciplinary team members, may help preserve continuity and provide holistic support. In addition, our data highlight the importance of providing enhanced support after discontinuation to address possible depressive feelings about treatment failure and help patients transition successfully to alternative regimens.

Based on our findings, we recommend four strategies to optimize LAI‐CAB/RPV implementation. First, clinics should consider injection support programmes featuring structured education, consistent staff members administering injections, comfortable environments and anticipatory guidance on pain management, possibly including peers. Second, multidisciplinary teams should provide clear, person‐centred guidance for managing late or missed doses, framed around partnership rather than surveillance. Third, clinics should preserve the continuity and connection with trusted providers, even as delivery becomes more decentralized. Fourth, clinics should utilize bundled visits as opportunities to assess patients’ evolving needs and provide additional support during periods of transition, including potential treatment discontinuation.

Some recommendations described here (e.g. consistent injection staffing, prescribing provider continuity or dedicated spaces to reduce injection‐related anxiety) may be difficult to implement in low‐ and middle‐income country (LMIC) settings. While differentiated service delivery [24], including integration of HIV care with non‐communicable disease (NCD) management [25], task shifting [26] and peer‐led approaches [27] are already embedded in many LMIC HIV programmes and may facilitate LAI‐ART integration, the frequency of injection visits, cold‐chain requirements and additional workforce demands risk exacerbating existing inequities or diverting critical resources from oral ART. Therefore, structured education and person‐centred communication [28], including peer support, contingency planning for missed doses and strategically bundled visits that leverage existing NCD infrastructure, may represent the most immediately feasible and scalable components of our recommendations for LMICs. Ultimately, implementation strategies must be locally adapted and, in some cases, fundamentally reimagined in partnership with local providers, communities and health systems to ensure the introduction of LAI‐ART is equitable, cost‐conscious and sustainable.

Our findings should be interpreted considering several limitations. The study population was drawn from urban academic medical centres, potentially limiting transferability to community clinics with fewer resources. In addition, the sample included relatively few younger adults and women, whose experiences and perceptions of LAI‐CAB/RPV may differ from those of other populations. Our discussion of combining LAI‐ART visits with primary care reflects experiences at sites where HIV care is integrated within primary care models. This approach may not be feasible or representative of all settings, particularly specialty or academic clinics that do not provide primary care services. Finally, interviews were conducted early in LAI‐CAB/RPV implementation and may not capture the experience and needs of PWH as these clinic programmes evolve.

## 5 CONCLUSIONS

Optimizing the LAI‐CAB/RPV patient journey requires more than technical precision, it calls for systems of care that are flexible, person‐centred, and attuned to both the practical and emotional dimensions of treatment. Across settings, this entails facilitating candid discussions about adherence contingencies, managing both the physical and psychological aspects of injection experiences, ensuring meaningful and consistent provider relationships amid changing care patterns and providing enhanced support during treatment transitions. As countries worldwide move towards long‐acting injectable ART options, these patient‐informed insights can help guide implementation approaches that are equitable, sustainable and compassionate.

## AUTHOR CONTRIBUTIONS

KAC, KAK and XAE designed the study. JG and JO were essential in the recruitment of patients who discontinued LAI‐CAB/RPV. MBS, PP, AD, XAE, KVD and RSW collected the data, and MBS, AD, XAE, KVD and RSW coded interview transcripts. KAC, MBS, PP, AD, XAE, KVD, RSW, JAC, LFC, MCM and KAK analysed the data, with MDH, ETM, MOJ, JS and JIG providing ongoing critical intellectual feedback. XAE wrote the paper. All authors have read and approved the final manuscript.

## FUNDING

This study was supported by R01 MH123396 (KAC). LFC is supported by K23 AG0844.

## COMPETING INTERESTS

The authors declare no competing interests.

## Data Availability

Data are not publicly available due to privacy concerns. Data from code excerpts are available on request from the corresponding author.
